# Skeletal muscle oxygenation in severe trauma patients during haemorrhagic shock resuscitation

**DOI:** 10.1186/s13054-015-0854-4

**Published:** 2015-04-06

**Authors:** Jerome Duret, Julien Pottecher, Pierre Bouzat, Julien Brun, Anatole Harrois, Jean-Francois Payen, Jacques Duranteau

**Affiliations:** Pole Anesthésie-Réanimation, Hôpital Michallon, Boulevard de la Chantourne, Grenoble, F-38043 France; Université Joseph Fourier, Grenoble Institut des Neurosciences, 6 rue Jules Horowitz, Grenoble, F-38043 France; INSERM, U836, Chemin Fortuné Ferrini, Grenoble, F-38042 France; Hôpitaux Universitaires de Strasbourg, Pôle Anesthésie Réanimation Chirurgicale SAMU, Hôpital de Hautepierre, Service d’Anesthésie-Réanimation Chirurgicale, 1 avenue Molière, F-67098 Strasbourg, France; Université de Strasbourg, Fédération de Médecine Translationnelle de Strasbourg (FMTS), Faculté de Médecine, Institut de Physiologie, Equipe d’Accueil EA3072 “Mitochondrie, stress oxydant et protection musculaire”, 11 rue Humann, F-67000 Strasbourg, France; AP-HP, Service d’ Anesthésie-Réanimation, Hôpitaux Universitaires Paris-Sud, Université Paris-Sud, Hôpital de Bicêtre, 78, rue du Général Leclerc, F-94275 Le Kremlin-Bicêtre, France; Laboratoire d’Etude de la Microcirculation, “Bio-CANVAS: Biomarqueurs in CardiaNeuroVascular Diseases” UMRS 942, 2 Rue Ambroise-Paré, 75010 Paris, France

## Abstract

**Introduction:**

Early alterations in tissue oxygenation may worsen patient outcome following traumatic haemorrhagic shock. We hypothesized that muscle oxygenation measured using near-infrared spectroscopy (NIRS) on admission could be associated with subsequent change in the SOFA score after resuscitation.

**Methods:**

The study was conducted in two Level I trauma centres and included 54 consecutive trauma patients with haemorrhagic shock, presenting within 6 hours of injury. Baseline tissue haemoglobin oxygen saturation (StO2) in the thenar eminence muscle and StO2 changes during a vascular occlusion test (VOT) were determined at 6 hours (H6) and 72 hours (H72) after the admission to the emergency room. Patients showing an improved SOFA score at H72 (SOFA improvers) were compared to those for whom it was unchanged or worse (SOFA non-improvers).

**Results:**

Of the 54 patients, 34 patients were SOFA improvers and 20 SOFA non-improvers. They had comparable injury severity scores on admission. SOFA improvers had higher baseline StO2 values and a steeper StO2 desaturation slope at H6 compared to the SOFA non-improvers. These StO2 variables similarly correlated with the intra-hospital mortality. The StO2 reperfusion slope at H6 was similar between the two groups of patients.

**Conclusions:**

Differences in StO2 parameters on admission of traumatic haemorrhagic shock were found between patients who had an improvement in organ failure in the first 72 hours and those who had unchanged or worse conditions. The use of NIRS to guide the initial management of trauma patients with haemorrhagic shock warrants further investigations.

## Introduction

Improvements in the early management of traumatic haemorrhagic patients have contributed to the reduction of mortality and morbidity over the last decades. Haemorrhage following traumatic injury accounts for 30 to 40% of deaths. But, after the control of the bleeding, major trauma patients continue to die in the ICU and multiple organ failure accounts for 20% of deaths [1].

Alterations of tissue perfusion and oxygenation due to an impaired microcirculation have been shown to contribute to the subsequent development of organ dysfunction and unfavourable outcome [2,3]. Such impaired microcirculation has been shown to last 72 hours despite controlled bleeding after traumatic haemorrhagic shock [4]. Early identification of microvascular hypoperfusion and inadequate resuscitation in trauma patients is necessary to prevent multiple organ dysfunction (MOD).

Extensive efforts have been made over the last few decades to identify markers of tissue oxygen deficit during the initial management of trauma patients in haemorrhagic shock. Low values of central venous oxygen saturation (ScvO2) and serum base excess (BE), and elevated values of arterial blood lactate on admission were found to correlate with poor outcome [5-7]. However, the information these markers provide of whole-body oxygen deficit may be too delayed to appreciate sufficiently early the severity of haemorrhagic shock compared to local measurements of tissue oxygenation [8].

Near-infrared spectroscopy (NIRS) is a non-invasive technology that allows the continuous measurement of tissue haemoglobin oxygen saturation (StO2) in the thenar eminence muscle [9,10]. In trauma patients, StO2 values at admission have been shown to correlate with ScvO2, BE and arterial lactate values [8], and with the severity of haemorrhagic shock [11]. Indeed, a low StO2 at the early phase of trauma was proposed to predict organ dysfunction and/or death [12,13]. In addition to static StO2 measurements at baseline, a forearm ischemia/reperfusion test can be applied in patients to obtain dynamic measures of StO2. The vascular occlusion test (VOT) assesses the response of the microvascular blood flow to a local hypoxic stimulus, in calculating the StO2 desaturation slope (DS-StO2) during transient ischemia and the StO2 recovery slope (RS-StO2) during a reactive hyperaemia [14]. The DS-StO2 and the RS-StO2 have been proposed as markers of tissue oxygen consumption and postischemic vasodilatation and capillary recruitment, respectively [15,16]. Septic patients with unfavourable outcome were shown to have a slower rate of StO2 recovery, as revealed by shallower slopes [17]. On the other hand, StO2 desaturation and recovery slopes became steeper along with improved haemodynamics in parturients admitted for postpartum haemorrhage [18]. In trauma patients, the DS-StO2 has been associated with need for in-hospital life-saving interventions [19].

In a cohort study of severe trauma patients with haemorrhagic shock, we searched for a possible association between StO2 changes during VOT on admission and the subsequent development of MOD as defined by degradation of the sequential organ failure assessment (SOFA) score.

## Methods

### Patient population

This prospective observational cohort study took place between April 2007 and April 2010 in two Level I French Trauma Centres: Grenoble and Bicêtre. The Institutional Review Board of Paris-Sud V approved the design of the study and waived the requirements for written informed consent from each patient (Comité de Protection des Personnes, Ile de France VII, A01226-47).

Adult patients were prospectively enrolled in the study if they were admitted to the emergency room (ER) within 6 hours of their blunt trauma. Inclusion criteria was the presence of haemorrhagic shock as defined by a systolic arterial blood pressure (SBP) of less than 90 mmHg despite the use of vasopressors and over 2 L crystalloid fluids, and the required transfusion of more than two units of packed red blood cells (PRBCs). Exclusion criteria were severe traumatic brain injury with initial Glasgow coma scale score of 3 or 4, cardiac arrest at initial presentation, moribund patients and those with a life expectancy of less than 24 h at their presentation to the ER and impossibility of using NIRS monitoring.

Each patient was mechanically ventilated to maintain normocapnia and normoxia. Sedation and analgesia were provided by continuous intravenous infusions of midazolam and sufentanil. Therapeutic decisions, such as use of haemostatic agents in surgery or requirement for angiographic arterial embolisation, were left to the discretion of the physician in charge of the patient. In accordance with our standard management of trauma patients, femoral venous and arterial lines were placed to monitor haemodynamics and allow transfusion of PRBCs and fresh frozen plasma (FFP) at a 1:1 ratio, as well as one unit of pooled platelets every six units of PRBC. Haemostatic disorders were corrected using fibrinogen concentrate and FFP if needed. The aim of this early phase of resuscitation in multiple trauma patients was to maintain arterial pressure, urine output ≥0.5 mL.kg-1.hr-1, blood lactate less than 2 mmoL/l, maintain haemoglobin content between 70 and 90 g/L, platelet count over 50 × 10^9^/L, plasma fibrinogen level over 1.0 g/L, activated partial thromboplastin time below 1.5 times control, and body temperature between 35 and 37°C, in accordance with European guidelines [20].

### Study protocol

StO2 was measured by a commercially available tissue spectrometer (InSpectra™ model 650, Hutchinson Technology, Hutchinson, MN, USA) linked to a probe that is placed on the thenar eminence muscle and contains two fibre-optic endings spaced 15 mm apart (Thenar Sensor™ model 1615, Hutchinson Technology, Hutchinson, MN, USA). The monitor operates at four discrete near-infrared optical wavelengths (680, 720, 760, and 800 nm) to provide continuous optical attenuation measurements of the tissue. In this range of optical wavelengths, oxyhaemoglobin and deoxyhaemoglobin (HbO2 and HbR), myoglobin, and oxidized cytochrome [21] display different light absorption spectra. The contribution of myoglobin and oxidized cytochrome to the light attenuation signal is negligible [9]. In addition to baseline measurements, StO2 was measured during a VOT test for which transient ischemia of the upper limb was induced by rapid inflation of a pneumatic cuff to a pressure 50 mmHg above the SBP for 3 minutes. The steady decline of thenar StO2 during this no-flow phase allowed the calculation of the DS-StO2 before pneumatic cuff release after which the RS-StO2 could be calculated. Both slopes were calculated from numerical values using the least-squares linear regression method. Only slopes with a linear correlation coefficient r^2^ of more than 0.90 were considered for analysis. Nadir StO2 values during VOT were also recorded.

Data were collected 6 h (H6) and 72 h (H72) following arrival in the ER (H0). They included patient demographics, injury severity score (ISS), simplified acute physiological score (SAPS II), clinical and biological variables, and StO2 measurements before and during VOT. Cardiac index (CI) was estimated from arterial pulse wave analysis using a commercially available transducer (FloTrac; Edwards Lifesciences, Irvine, CA, USA) and monitor (Vigileo; Edwards Lifesciences, Irvine, CA, USA).

### Statistical analysis

According to changes in their SOFA score, patients were dichotomized for the analysis into those with an improved SOFA score at H72 (SOFA improvers) and those with an unchanged or worsened SOFA score at H72 (SOFA non-improvers). An improved SOFA score at H72 was defined as a delta SOFA from H6 to H72 <0. Unchanged or worsened SOFA score at H72 was defined as a delta SOFA score ≥0. Patients who died after the 24^th^ hour of MOD were considered as SOFA non-improvers. Other analyses included intra-hospital survival rate, and the treatments administered throughout the study period. Descriptive statistics included frequencies and percentages for categorical variables and the median (extremes) for continuous variables. The StO2 slopes were expressed as a percentage change in StO2 per minute. Comparisons were made using non-parametric Mann-Whitney or Wilcoxon tests and the Fisher’s exact test where appropriate (Prism 5.0, GraphPad Software, La Jolla, CA, USA). A *P* value of 0.05 or less was considered statistically significant.

## Results

Fifty-four severe trauma patients with haemorrhagic shock were included in the study: 24 from Grenoble and 30 from Bicêtre Level I trauma centres. Twelve patients died during their ICU stay after the control of the bleeding and after the 24^th^ hour. All these patients died of MODs. One patient among the SOFA improvers died on day 14 while in the SOFA non-improvers nine patients died before the 72^th^ hour and two died after the 72^th^ hour (day 6 and day 8 respectively). The SOFA score at H72 improved in 34 patients (SOFA improvers) and either remained unchanged or had worsened in the remaining 20 patients (SOFA non-improvers). The two groups had comparable ISS on admission. However, the intra-hospital mortality rate was significantly reduced among SOFA improvers (Table 1). The two groups of patients showed no difference at H6 with regards requirements for vasopressors, fluids and blood products (Table 2). The SOFA improvers had a higher CI, a lower BE deficit and a lower arterial blood lactate, and less frequently presented haemostatic disorder during their initial management.

**Table 1:** **Demographic data (n or median [25** ^**th**^ **to 75** ^**th**^ **percentiles]) collected from the 54 haemorrhagic trauma patients on their admission to the emergency room**

	**SOFA improvers**	**SOFA non-improvers**
	**(n = 34)**	**(n = 20)**
BMI (kg/m^2^)	23.7 [22.0-25.8]	24.7 [23.6-26.3]
Sex ratio (M/F)	22/12	16/4
Injury type (n)		
Road accident	22	8
Fall	10	7
Other	2	5
Injury to admission period (min)	60 [35-300]	70 [30-180]
ISS	27 [20-42]	34 [25-47]
SAPS II	43 [17-77]	61 [28-105]^*^
Control of bleeding (n)		
Haemostatic surgery	19	7
Embolisation	3	4
Both	10	5
Nothing	2	4
SOFA score at H6	11 [8-13]	13 [9-16]^*^
SOFA score at H72^a^	6 [3-9]	16 [10-23]^*^
Delta SOFA score between H72 and H6^a^	-4 [-2--7]	2 [8-0]^*^
Intra-hospital mortality (n)	1	11^*^

Patients were dichotomised according to their SOFA score changes at H72. SOFA score measured at H72 was either improved if the delta SOFA score between H72 and H6 was <0 (SOFA improvers; n = 34 patients), or unchanged or aggravated SOFA score if the delta SOFA score was ≥0 (SOFA non-improvers; n = 20 patients). ^a^Nine patients died between H24 and H72 in SOFA non-improvers. *P <0.05 vs SOFA improvers. BMI, body mass index; ISS, injury severity score; SAPS, simplified acute physiological score; SOFA, sequential organ failure assessment.

**Table 2:** **Physiological variables (n or median [25** ^**th**^ **to 75** ^**th**^ **percentiles]) of the 54 trauma patients with haemorrhagic shock collected 6 hours (H6) after their arrival at the emergency room**

	**SOFA improvers**	**SOFA non-improvers**
	**(n = 34)**	**(n = 20)**
SBP (mmHg)	97 [91-118]	101 [71-109]
Heart rate (bpm)	117 [96-125]	110 [87-119]
CI (l/min/m^2^)	3.2 [2.5-3.8]	2.5 [1.8-2.8]^*^
Temperature (°C)	36.2 [35.7-36.8]	35.6 [33.6-36.4]^*^
Urine output (mL/h)	30 [10-100]	25 [0-158]
Use of vasopressors (n)	31	18
Arterial blood lactate (mmoL/L)	3.9 [2.5-5.8]	5.4 [3.0-12]^*^
BE deficit (mmoL/L)	−7.2 [-11.7--4.5]	−11.4 [-17--7]^*^
Arterial pH	7.29 [7.21-7.36]	7.16 [7.03-7.37]
Haemoglobin (g/L)	84 [73-108]	91 [76-99]
Platelets (G/L)	149 [122-225]	118 [99-150]^*^
Activated PTT (sec)	39 [35-43]	40 [35-120]^*^
PRBC (units)	7 [5-9]	8 [5-12]
FFP (units)	6 [4-8]	7 [5-10]
Cristalloids (mL)	2,000 [1,000-3,000]	1,500 [1,500-2,713]
Colloids (mL)	1,500 [1,500-2,125]	1,625 [1,500-2,500]

Patients were separated into two groups according to whether they subsequently improved or not their initial SOFA score (see definition Table 1). *P <0.05 vs SOFA improvers. SBP, systolic blood pressure; CI, cardiac index; BE, base excess; PTT, partial thromboplastin time; PRBCs, packed red blood cells; FFP, fresh frozen plasma; SOFA, sequential organ failure assessment.

**Figure 1:**
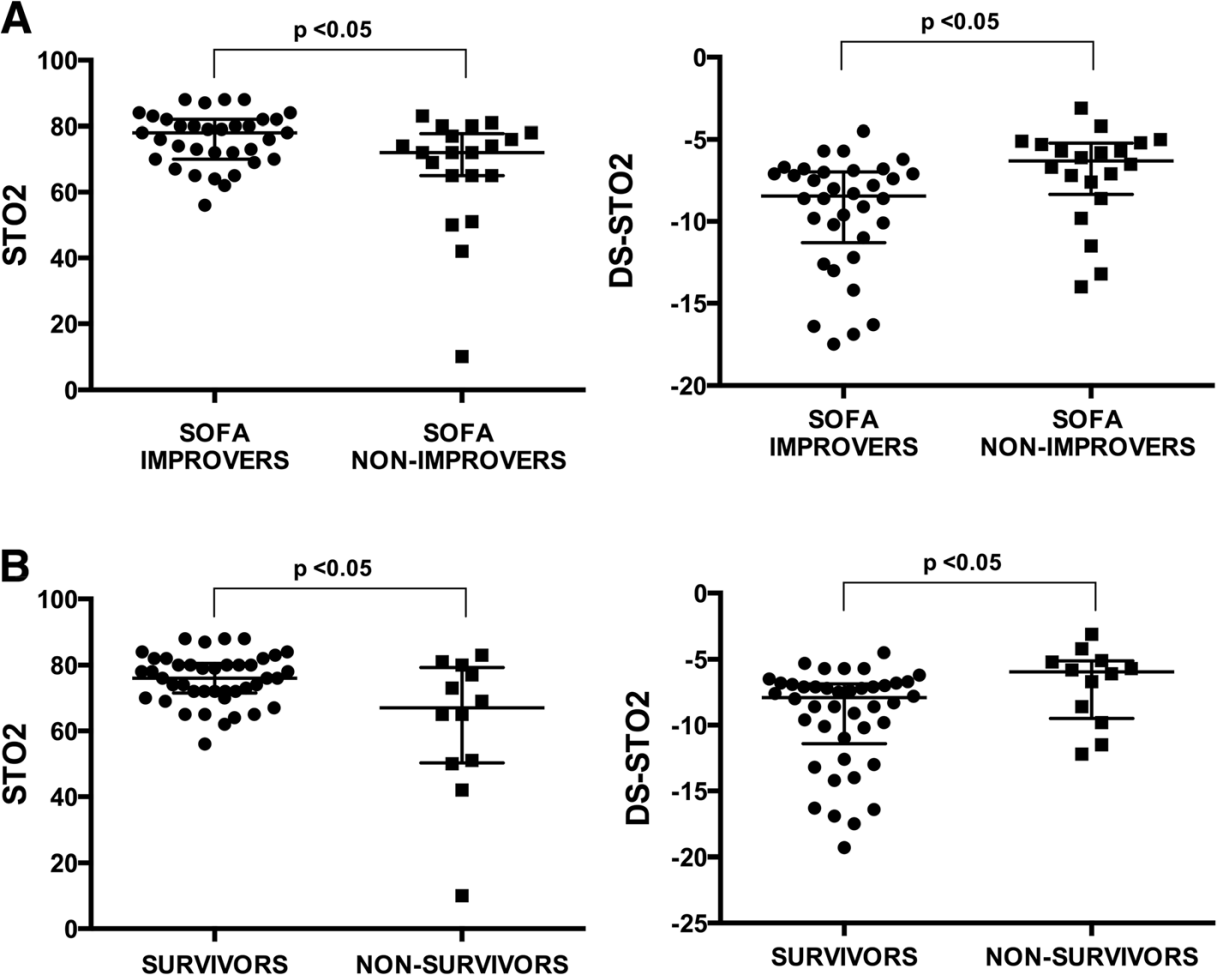
**Scatter dot plot (median with interquartile range) of StO2 measurements (baseline and StO2 desaturation slope) collected 6 hours after admission (H6) from the 54 trauma haemorrhagic patients.** Patients were dichotomised according to their SOFA score at H72 **(A)** and to the in-hospital mortality **(B)**. Improved SOFA score at H72 was defined as a delta SOFA score <0 (SOFA improvers, n = 34 patients), and unchanged or aggravated SOFA score at H72 by a delta SOFA score ≥0 (SOFA non-improvers, n = 20 patients). *P <0.05 vs. SOFA improvers or survivors. StO2, tissue haemoglobin oxygen saturation; SOFA, sequential organ failure assessment.

The SOFA improvers had higher baseline StO2 values and a steeper DS-StO2at H6 compared to the SOFA non-improvers (Table 3). Use of intra-hospital mortality as a criterion gave similar results (Figure 1A and B). SOFA improvers and SOFA non-improvers displayed similar StO2 reperfusion slopes at H6. In survivors of each group, the baseline StO2 and the StO2 desaturation slope at H72 were improved with respect to that measured at H6 (*P* <0.05). No differences were observed at H72 between SOFA improvers and SOFA non-improvers about the baseline StO2, the DS-STO2 and the RS-StO2. The two groups showed no difference with regards duration of mechanical ventilation: 6 days [1; 28 days] among SOFA improvers versus 2 days [1; 42 days] among SOFA non-improvers, respectively. They also stayed for comparable length of time in the ICU: 12 days [1; 50] for SOFA improvers versus 6 days [1; 52] for non-improvers.

**Table 3:** **StO2 values (median [25** ^**th**^ **to 75** ^**th**^ **percentiles]) of the 54 trauma patients with haemorrhagic shock collected 6 hours (H6) and 72 hours (H72) after their arrival at the emergency room**

	**SOFA improvers**	**SOFA non-improvers**
	**(n = 34 patients)**	**(n = 20 patients)**
Baseline StO2 at H6 (%)	78 [70-82]	72 [65-78]^*^
Nadir StO2 during VOT at H6 (%)	49 [45-58]	52 [37-60]
DS-StO2 at H6 (%/min)	−8.4 [-11.3--6.97]	−6.6 [-9.5--5.4]^*^
RS-StO2 at H6 (%/min)	78.0 [60.5-125.8]	62.2 [42.4-94.2]
Baseline StO2 at H72 (%)^a^	87 [81-90]	84 [78-86]
Nadir StO2 during VOT at H72 (%)^a^	54 [49-61]	60 [55-68]
DS-StO2 at H72 (%/min)^a^	−10.5 [-12.9--8.9]	−9.1 [-10.5--7.6]
RS-StO2 at H72 (%/min)^a^	147.8 [89.3-201.7]	93.5 [72-168]

Patients were separated into two groups according to whether they subsequently improved or not their initial SOFA score (see definition Table 1). ^a^Nine patients died between H24 and H72 in SOFA non-improvers. *P <0.05 vs SOFA improvers. StO2, tissue haemoglobin oxygen saturation; SOFA, sequential organ failure assessment; VOT, vascular occlusion test; DS-StO2, StO2 desaturation slope; RS-StO2, StO2 slope.

## Discussion

Data from our prospective cohort of trauma patients with haemorrhagic shock admitted to the ER of two Level I trauma centres indicate that muscular StO2 measured 6 hours after arrival is associated with subsequent change in SOFA score. Specifically, despite comparable ISS on admission, both baseline StO2 and DS-StO2 significantly differed between patients in whom the SOFA score improved and those for whom it did not after bleeding control and haemodynamic stabilization.

To assess muscle StO2, we used NIRS to measure the ratio of oxygenated and deoxygenated haemoglobin. While some uncertainty surrounds use of NIRS for cerebral measurements [22], muscle thenar StO2 measurements are negligibly influenced by skin and other tissues [9]. During the resuscitation of trauma patients, low StO2 values at baseline and high BE deficit have been associated with the development of MOD and death [7,12,13]. In line with these results, we found associations between these two parameters on admission and subsequent change in SOFA score, the development of MOD at H72 and the risk of death.

One study had previously tested dynamic StO2 measurements during the prehospital phase of trauma patients [19]. In that study, a decreasing in DS-StO2 was associated with a higher proportion of patients requiring in-hospital life-saving interventions, that is, blood transfusion, thoracotomy, laparotomy, pelvic fixation, and embolisation. In line with that study, we found that the DS-StO2 was significantly less steep on admission in SOFA non-improvers.

The rate of StO2 desaturation during ischemic no-flow challenge has been proposed to estimate muscle oxygen consumption [23] and be steeper during exercise than at rest [15]. The DS-StO2 can be decreased as a consequence of a lower tissue metabolic rate due to an impaired regional perfusion and/or to a decrease in tissue oxygen consumption. A reduction in tissue oxygen consumption could be due to processes of cell adaptation to hypoxia or to an early impairment of mitochondrial oxidative function induced by haemorrhagic shock [24,25]. Previous studies have shown that the DS-StO2 is predictive of poor outcomes [14,17]. A decrease in DS-StO2 was found in patients with septic shock [26,27] and was correlated with the patient outcome [26]. Changes in oxidized cytochrome have also been shown to outweigh those in oxyhaemoglobin in trauma patients who subsequently developed MODS [28]. In addition, this dynamic parameter was found to improve in parturients after haemodynamical stabilisation and bleeding control [18]. Collectively, our findings suggest that in severe trauma patients with haemorrhagic shock who showed no improvement in SOFA score might have suffered an early impairment in muscle tissue oxygen consumption. Of note were higher BE deficit and higher arterial blood lactate in the SOFA non-improvers. Elevation of blood lactate reflects flow-demand mismatch as a consequence of haemorrhagic shock. Its persistence despite control of haemorrhage suggests needs for more fluid resuscitation. Conversely, the DS-StO2 may provide information about the tissue oxygen consumption. A reduction in tissue oxygen consumption as reflected by a decreasing in the DS-StO2 can be due to the processes of cell adaptation to hypoxia (oxygen conformance) and/or to an early impairment of mitochondrial oxidative function. These two mechanisms are involved in organ dysfunction [29]. In addition, blood lactate is a systemic marker while StO2 and its changes during a VOT explore regional tissue perfusion. StO2 parameters can detect persistent occult tissue hypoperfusion.

The RS-StO2 is assumed to reflect the capacity of microvessels to vasodilate and/or to be recruited in response to a local hypoxic stimulus [23,26,30]. In healthy volunteers, the RS-StO2 after ischemic challenge has been shown, by variations in muscle magnetic resonance imaging (MRI) signal intensity, to correlate with changes in local muscle blood volume [31]. Ischemic stimulus induces the dilation of precapillary arterioles, the opening of closed capillaries (recruitment), and the increase in tissue blood flow in previously patent capillaries, a phenomenon commonly called ‘reactive hyperaemia’. Accordingly, the RS-StO2 should reflect the ability of microvessels to be recruited in response to a local hypoxic stimulus. Creteur *et al*. reported a slower RS-StO2 among a group of non-survivors who suffered septic shock [17]. The sepsis-induced decrease in microvascular capillary density would limit the number of recruitable capillaries following an ischemic challenge, and thus alter the reactive hyperaemia [32]. Trauma patients have also been shown to have a shallower RS-StO2 than normal volunteers [14], indicating that trauma induces pre-existing tissue vasoconstriction that could be unmasked by the VOT. The lack of differences in the early measurements of RS-StO2 between our two groups of patients suggests that local changes in the capillary recruitment following haemorrhagic shock played a moderate role in the subsequent development of organ failure. When comparing with the RS-StO2, the DS-StO2 is more the result of a cellular process in response to hypoperfusion and/or hypoxia. The RS-StO2 reflects more the tissue hypoperfusion and the decrease in red blood concentration. The DS-StO2 reflects a cellular process in response to hypoperfusion and/or hypoxia. The RS-StO2 is believed to reflect the capacity of microvessels to vasodilate. A transient tissue hypoperfusion could be tolerated by cells with no change in tissue oxygen metabolism or organ dysfunction. It is then reasonable to consider that organ dysfunction might be more accurately reflected by changes in the DS-StO2. However, interpreting the RS-StO2 data should be cautious due to the use of a 3-min cuff inflation procedure as ischemic challenge. This procedure was further found to be less accurate than cuff inflation until muscle StO2 decreased to 40% [33]. It might be possible that our ischemic challenge was not sufficiently low to trigger a complete microcirculatory response.

Another limit of the study is the fact that neither multivariate analysis nor a receiver operating characteristic curve with calculation of sensitivity and specificity could be performed, due to the limited number of patients. Although we obtained consistent results between both centres, evaluation of the diagnostic performance of StO2 measurements would have required a larger population of patients.

## Conclusions

Differences in StO2 parameters on admission of traumatic haemorrhagic shock were found between patients who had an improvement in organ failure in the first 72 h and those who had unchanged or worse conditions. The use of NIRS to guide the initial management of trauma patients with haemorrhagic shock warrants further investigations.

## Key messages

- It is feasible to obtain information about the microcirculation using tissue haemoglobin oxygen saturation (StO2) measurements with near-infrared spectroscopy (NIRS) during the early phase of resuscitation of trauma patients with haemorrhagic shock.
- Differences in StO2 parameters on admission of traumatic haemorrhagic shock were found between patients who had an improvement in organ failure in the first 72 h and those who had unchanged or worse conditions.
- The use of NIRS to guide the initial management of trauma patients with haemorrhagic shock warrants further investigations.

## Abbreviations

BE: base excess
CI: cardiac index
DS-StO2: StO2 desaturation slope
ER: emergency room
FFP: fresh frozen plasma
H6: 6 hours
H72: 72 hours
HbO2: oxyhaemoglobin
HbR: deoxyhaemoglobin
ISS: injury severity score
MRI: magnetic resonance imaging
MOD: multiorgan dysfunction
NIRS: near-infrared spectroscopy
PRBCs: packed red blood cells
RS-StO2: StO2 slope
SAPS: simplified acute physiological score
SBP: systolic blood pressure
ScvO2: central venous oxygen saturation
SOFA: sequential organ failure assessment
StO2: tissue haemoglobin oxygen saturation
VOT: vascular occlusion test
